# Practice patterns for postoperative radiation therapy in patients with metastases to the long bones: a survey of the Japanese Radiation Oncology Study Group

**DOI:** 10.1093/jrr/rraa133

**Published:** 2021-01-18

**Authors:** Hikaru Kubota, Naoki Nakamura, Naoto Shikama, Ayako Tonari, Hitoshi Wada, Hideyuki Harada, Hisayasu Nagakura, Joichi Heianna, Kei Ito, Miwako Nozaki, Masao Tago, Masato Fushiki, Nobue Uchida, Norio Araki, Shuhei Sekii, Takashi Kosugi, Takeo Takahashi, Terufumi Kawamoto, Tetsuo Saito, Kazunari Yamada

**Affiliations:** Department of Radiation Oncology, Kobe University Hospital, 7-5-2 Kusunoki-cho, Chuo-ku, Kobe City, Hyogo Prefecture 650-0017, Japan; Department of Radiation Oncology, St. Marianna University School of Medicine, 2-16-1 Sugao Kawasaki City, Kanagawa prefecture 216-8511, Japan; Department of Radiation Oncology, Juntendo University Hospital, 3-1-3 Hongo, Bunkyo-ku, Tokyo 113-8431, Japan; Department of Radiation Oncology, Kyorin University Hospital, 6-20-2 Shinkawa, Mitaka-shi, Tokyo 181-8611, Japan; Department of Radiation Oncology, Southern TOHOKU Proton Therapy Center, 172-7, Yatsuyamada,Koriyama,Fukushima 963-8563, Japan; Division of Radiation Therapy, Radiation and Proton Therapy Center, Shizuoka Cancer Center Hospital, 1007 Shimonagakubo, Nagaizumi-cho, Sunto-gun, Shizuoka Prefecture 411-8777, Japan; Department of Radiology, KKR Sapporo Medical Center, 6-3-40 Hiragishi-1, Toyohira-ku, Sapporo 062-0931, Japan; Department of Radiation Oncology, Ryukyu University Hospital; Department of Radiation Oncology, Tokyo Metropolitan Cancer and Infectious Diseases Center Komagome Hospital, 3-18-22 Honkomagome, Bunkyo-ku, Tokyo 113-8677, Japan; Department of Radiation Oncology, Dokkyo Medical University Saitama Medical Center, 2-1-50 Minamikoshigaya, Koshigaya, Saitama, Japan; Department of Radiology, Teikyo University Mizonokuchi Hospital, 3-8-3, Mizokuchi, Takatsu-ku, Kawasaki city, Kanagawa pref., Japan; Department of Radiation Oncology, Nagahama City Hospital, 313 Oinuicho, Nagahama, Shiga 526-8580, Japan; Department of Radiation Oncology, Tokyo Saiseikai Central Hospital, 1-4-17 Mita, Minato-ku, Tokyo 108-0073, Japan; Department of Radiation Oncology, Kyoto Medical Center, 1-1 Fukakusamukaihatacho, Fushimi-ku, Kyoto-shi, Kyoto 612-8555, Japan; Department of Radiation Oncology, Kobe University Hospital, 7-5-2 Kusunoki-cho, Chuo-ku, Kobe City, Hyogo Prefecture 650-0017, Japan; Department of Radiation Oncology, Fujieda Municipal General Hospital, 4-1-11 Surugadai, Fujieda City, Shizuoka Prefecture 426-8677, Japan; Department of Radiation Oncology, Saitama Medical University Saitama Medical Center, 1981 kamoda,kawagoeshi, saitama 350-8550, Japan; Department of Radiation Oncology, Juntendo University Hospital, 3-1-3 Hongo, Bunkyo-ku, Tokyo 113-8431, Japan; Department of Radiation Oncology, Arao Municipal Hospital, 2600 Arao, Arao City, Kumamoto 864-0041, Japan; Department of Radiation Oncology, Seirei Mikatahara General Hospital, 3453 Mikatahara-cho, Kita-ku, Hamamatsu, Shizuoka 433-8558, Japan

**Keywords:** bone metastases, long bones, postoperative radiation therapy, patterns of practice, oligometastasis

## Abstract

Evidence regarding postoperative radiation therapy (PORT) for metastases to the long bones is lacking. Characterizing the current practice patterns and identifying factors that influence dose-fractionation schedules are essential for future clinical trials. An internet-based survey of the palliative RT subgroup of the Japanese Radiation Oncology Study Group was performed in 2017 to collect data regarding PORT prescription practices and dose-fractionation schedules. Responders were also asked to recommend dose-fractionation schedules for four hypothetical cases that involved a patient with impending pathological fractures and one of four clinical features (poor prognosis, solitary metastasis, radio-resistant primary tumor or expected long-term survival). Responders were asked to indicate their preferred irradiation fields and the reasons for the dose fractionation schedule they chose. Responses were obtained from 89 radiation oncologists (67 institutions and 151 RT plans) who used 22 dose-fractionation schedules, with the most commonly used and recommended schedule being 30 Gy in 10 fractions. Local control was the most common reason for preferring longer-course RT. High-dose fractionated schedules were preferred for oligometastasis, and low-dose regimens were preferred for patients with a poor prognosis; however, single-fraction RT was not preferred. Most respondents recommended targeting the entire orthopedic prosthesis. These results indicated that PORT using 30 Gy in 10 fractions to the entire orthopedic prosthesis is preferred in current Japanese practice and that single-fraction RT was not preferred. Oligometastasis and poor prognosis influenced the selection of high- or low-dose regimens.

## INTRODUCTION

Bone metastases (BMs) are a frequent complication of cancer [1–3] and cause variable symptoms such as pain, pathological fractures and spinal cord compression. These symptoms reduce the patient’s quality of life and performance status. Radiation therapy (RT) is recognized as a highly effective standard therapy for BMs, as randomized trials have revealed that it provides pain relief to ~60–80% of patients [4]. Meanwhile, real-world evidence has revealed a slightly lower response rate (55%) [5].

Surgical interventions are often required to stabilize the long bones with impending or existing pathological fractures in cases of BM, and these interventions may provide pain relief and restore functional status [6]. The surgical strategies generally involve endoprosthetic reconstruction or internal fixation with either intramedullary nailing or plate/screw fixation devices. However, the indication for postoperative RT (PORT) after endoprosthetic reconstruction remains unclear, as PORT reportedly prevents new bone formation in cases of proximal femur metastasis [7]. Nevertheless, PORT is commonly used for metastases to the long bones, as a few retrospective studies have indicated that it reduces pain, improves functional status, decreases reoperation and slows the local progression of metastases [8–11]. However, the optimal dose-fractionation schedule and irradiation field have not been defined for PORT in this setting, and clinical trials will be needed to provide clear evidence to address these issues. The design of such trials must consider current decision-making practices for radiation oncologists as well as the existing standards of care, but studies regarding practice patterns for using PORT to treat metastases to the long bones are lacking. Therefore, the present study aimed to characterize the current Japanese practice patterns for using PORT to treat metastases to the long bones and to identify factors that affected the dose-fractionation schedule selection.

## MATERIALS AND METHODS

An internet-based survey of the palliative RT subgroup of the Japanese Radiation Oncology Study Group (JROSG) was performed in 2017 to collect data from practicing radiation oncologists. All respondents consented to participate in this survey. The respondents were asked to provide their name, institution, years of experience in radiation oncology practice, frequency of PORT use at each institution, and the indications and dose-fractionation schedules for using PORT to treat metastases to the long bones. Furthermore, the survey asked the respondents to recommend dose-fractionation schedules for four hypothetical cancer cases.

Each hypothetical case involved impending BM-related pathological fracture in the lower limb with one of four unique clinical features (Table 1). Case 1 involved a patient with poor expected survival who had an impending pathological fracture in the lower limb that was related to multiple metastases from non-small-cell lung cancer. Case 2 involved a patient with oligometastasis, and whether the management strategy for a solitary BM would be different from that in case 1 had to be determined. Case 3 involved a radio-resistant primary tumor (renal cell carcinoma, RCC) and was otherwise identical to case 2. Case 4 involved a patient with good expected long-term survival who had multiple BMs from breast cancer.

**Table 1:** Hypothetical cases

Case 1	Patients with relatively limited survival
	65-year-old man with squamous cell lung cancer had been treated by radical surgery 1 year earlier. Patient had right femoral pain, and examination shows lytic BM in right femoral bone, multiple lung metastases and right adrenal met. Internal fixation was performed for right femoral BM due to the impending fracture. He now has a little pain. His ECOG PS^a^ is 1.
Case 2	Patient with oligometastasis
	65-year-old man with squamous cell lung cancer had been treated by radical surgery 1 year earlier. Patient had right femoral pain, and examination shows solitary lytic BM in right femoral bone. Internal fixation was performed for right femoral BM due to the impending fracture. He now has a little pain. His ECOG PS is 1.
Case 3	Patient with the radio-resistant primary tumor
	65-year-old man with renal cell carcinoma had been treated by radical surgery 1 year earlier. Patient had right femoral pain, and examination shows lytic BM in right femoral bone and multiple lung metastases. Internal fixation was performed for right femoral BM due to the impending fracture. He now has a little pain. His ECOG PS is 1.
Case4	Patient with expected long survival
	50-year-old woman had right femoral pain. Examination showed left breast tumor and multiple lytic lesions including right femoral bone. She was diagnosed as breast cancer (ER positive/PR positive/Her-2 negative/Ki-67 5%) and multiple BMs. Internal fixation was performed for right femoral BM due to the impending fracture. She now has a little pain. Her ECOG PS is 2.

^a^ECOG PS (Eastern Cooperative Oncology Group performance status) is an indicator of the general condition, and clinical assessment is needed if activity is restricted by local symptoms.

The actual prescribed dose-fractionation schedules and the hypothetically recommended schedules were classified into five categories based on the biologically effective dose (BED10) (Table 2): ‘single fraction’ involved a dose of 14.4 Gy (BED10), ‘fractionated low dose’ involved a dose of 22.5–37.5 Gy (BED10), ‘30 Gy in 10 fractions’ involved a dose of 39 Gy (BED10), ‘fractionated intermediate dose’ involved a dose of 39.2–59.5 Gy (BED10) and ‘fractionated high dose’ involved a dose ≥60 Gy (BED10). Radiation oncologists who did not recommend 8 Gy in 1 fraction or 20 Gy in 5 fractions for case 1 were asked the reason for choosing such options. Recommendations regarding the irradiation field were classified as the ‘preoperative tumor location’, ‘entire orthopedic prosthesis’, or ‘entire affected bone’.

**Table 2:** Classification of dose-fractionations and prescribed dose per fraction used in each institution and recommended for hypothetical cases

	Dose-fractionation schedule	Dose per fraction (Gy)	BED10
Single fraction			
	8 Gy/1 fr^a^	8	14.4
Fractionated low dose			
	15 Gy/3 fr	5	22.5
	20 Gy/5 fr	4	28
	20 Gy/4 fr	5	30
	24 Gy/6 fr	4	33.6
	25 Gy/5 fr	5	37.5
30 Gy in 10 fractions			
	30Gy/10fr	3	39
Fractionated intermediate dose			
	28 Gy/7 fr	4	39.2
	24 Gy/3 fr	8	43.2
	36 Gy/12 fr	3	46.8
	37.5 Gy/15 fr	2.5	46.88
	30 Gy/5 fr	6	48
	40 Gy/16 fr	2.5	50
	39 Gy/13 fr	3	50.7
	42 Gy/14 fr	3	54.6
	40 Gy/10 fr	4	56
	45 Gy/18 fr	2.5	56.25
	45 Gy/15 fr	3	58.5
	35 Gy/5 fr	7	59.5
Fractionated high dose			
	50 Gy/25 fr	2	60
	48 Gy/12 fr	4	67.2
	60 Gy/30 fr	2	72
	50 Gy/10 fr	5	75
	60 Gy/24 fr	2.5	75
	65 Gy/25 fr	2.6	81.9

^a^fr = fraction.

## RESULTS

**Fig. 1.**
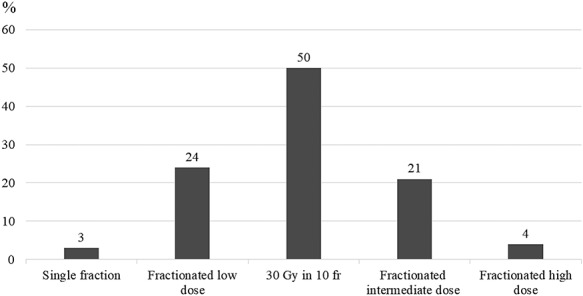
Dose-fractionation regimens used at the Japanese Radiation Oncology Study Group institutions. Single fraction: 14.4 Gy (BED10); fractionated low dose: 22.5–37.5 Gy; 30 Gy in 10 fractions: 39 Gy (BED10); fractionated intermediate dose: 39.2–59.5 Gy; fractionated high dose: ≥60 Gy.

Responses were obtained from 89 radiation oncologists (26% of the JROSG members) at 67 institutions (34% of the JROSG facilities). The responders’ median experience in radiation oncology was 20 years (range, 2.5–40 years). A total of 152 RT plans were used at these institutions in 2017, but one plan was excluded because of an undefined dose-fractionation schedule. The 151 RT plans included 21 dose-fractionation schedules, which ranged from 8 Gy in 1 fraction to 65 Gy in 25 fractions (supplementary Table 1, see online supplementary material). Single-fraction RT was prescribed in only 3% of patients (*n* = 4), and fractioned RT was prescribed in the remaining 97% of the patients (*n* = 147). Among the five dose-fractionation categories, the most common schedule was 30 Gy in 10 fractions (*n* = 75, 50%), which was followed by ‘fractionated low-dose’ schedules (Fig. 1). The most common ‘fractionated low-dose’ schedule involved 20 Gy in 5 fractions (*n* = 29, 19%).

**Fig. 2.**
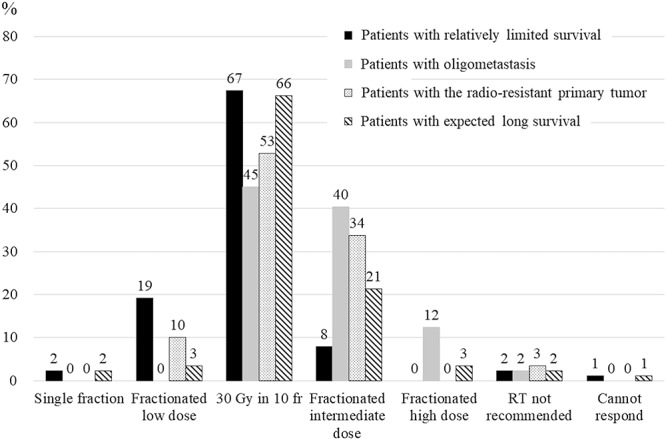
Dose-fractionation regimens recommended for hypothetical cases. The black bar represents case 1 (relatively limited survival), the gray bar represents case 2 (presence of oligometastasis), the dotted bar represents case 3 (radio-resistant primary tumor), and the hatched bar represents case 4 (expected long survival). Single fraction: 14.4 Gy (BED10); fractionated low dose: 22.5–37.5 Gy; 30 Gy in 10 fractions: 39 Gy (BED10); fractionated intermediate dose: 39.2–59.5 Gy; fractionated high dose: ≥60 Gy.

**Fig. 3.**
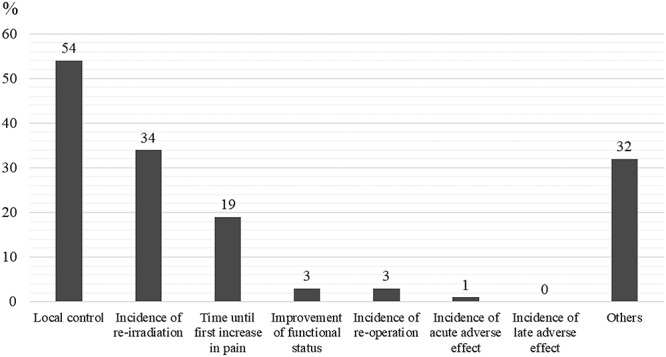
Respondents’ reasons for not recommending 8 Gy in 1 fraction or 20 Gy in 5 fractions for hypothetical case 1. Multiple response were allowed for this question. A total of 68 respondents recommended longer-course radiation therapy for case 1.

**Fig. 4.**
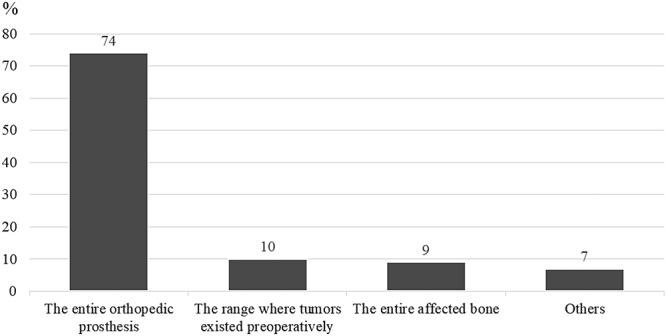
Recommended irradiation fields for postoperative radiation therapy.

The responders recommended 15 dose-fractionation schedules for the four hypothetical cases, which ranged from 8 Gy in 1 fraction to 60 Gy in 30 fractions (supplementary Table 2, see online supplementary material). For all four cases, the most common dose-fractionation schedule recommendation involved 30 Gy in 10 fractions (Fig. 2). For case 1, only 17 respondents (19%) recommended fractionated low-dose regimens and 68 respondents (76%) did not recommend 8 Gy in 1 fraction or 20 Gy in 5 fractions. Figure 3 summarizes the reasons of the responders for not recommending 8 Gy in 1 fraction or 20 Gy in 5 fractions to treat case 1. ‘Local control’ was the most common reason (*n* = 37, 54%), which was followed by ‘incidence of re-irradiation’ (*n* = 23, 34%) and ‘time until first increase in pain’ (*n* = 13, 19%). Fractionated dose regimens (>30 Gy in 10 fractions) were more commonly recommended for oligometastasis (*n* = 47, 53%) than for patients with a radio-resistant tumor (*n* = 30; 34%) or expected good long-term survival (*n* = 22, 25%). Fractionated intermediate-dose regimens were commonly recommended for oligometastasis (case 2) or a radio-resistant primary tumor (case 3). Fractionated high-dose regimens were recommended for oligometastasis (*n* = 11, 12%) but not for a radio-resistant primary tumor. A total of 59 respondents (66%) recommended 30 Gy in 10 fractions for case 4 (expected long-term survival), which was similar to the recommendation for case 1 (expected poor survival; *n* = 60, 67%). Most responders recommended that the PORT irradiation field involve the ‘entire orthopedic prosthesis’ (*n* = 66, 74%), which was followed by the ‘preoperative tumor location’ (*n* = 9, 10%) and ‘entire affected bone’ (*n* = 8, 9%) (Fig. 4).

## DISCUSSION

The present study revealed that, in Japan, 30 Gy in 10 fractions was the most common PORT schedule (~50% of all RT plans) for treating metastases to the long bones. In addition, most respondents recommended this regimen for the hypothetical cases (67% for case 1, 45% for case 2, 53% for case 3 and 66% for case 4). These results are similar to the results from previous retrospective studies regarding PORT for metastases to the long bones [8–11], which generally involved fractioned RT (e.g. 30 Gy in 10 fractions or 20 Gy in 5 fractions) and total doses of 8–56 Gy. The American College of Radiology Appropriateness Criteria for Non-Spine BMs [12], which are based on multidisciplinary expert opinions, do not provide a definitive recommendation regarding the most appropriate RT dose, but they indicate that 30 Gy in 10 fractions seems reasonable for eradicating microscopic residual disease. Expert opinions also indicate that schedules of 8 Gy in 1 fraction to 35 Gy in 14 fractions are also equally appropriate options.

In the present study, hypothetical case 2 involved a single oligometastasis of non-small-cell lung cancer in a long bone and had the strongest recommendation for a higher-dose regimen (fractionated intermediate dose, 40%, or fractionated high-dose, 12%), relative to the other hypothetical cases. The Stereotactic Ablative Radiotherapy for the Comprehensive Treatment of Oligometastatic Tumors study [13] recently revealed that stereotactic body RT (SBRT) for oligometastasis provided a survival benefit in terms of median overall survival (standard of care, 28 months vs SBRT, 41 months). Another study [14] also revealed that high-dose SBRT provided a high rate of local control (>85%) for non-spine BMs. Thus, high-dose PORT regimens might provide good local control and survival benefits in cases that involve oligometastasis.

Hypothetical case 3 involved a radio-resistant primary tumor but was otherwise identical to case 1, and none of the respondents recommended a high dose per fraction (>5 Gy/fraction) for case 3. Although RCC is traditionally considered radio-resistant [15], a high-dose per RT fraction may help overcome the relative radio-resistance of RCC [16]. Similar findings for melanoma have also been reported, which is also considered a radio-resistant primary tumor [17]. However, Rades *et al*. [18] have reported that dose escalation to > 30 Gy in 10 fractions did not significantly improve the motor function outcomes and local control of metastatic spinal cord compression in cases that involved radio-resistant tumors, such as RCC, colorectal cancer and malignant melanoma. Therefore, it remains unclear whether to escalate the radiation dose for radio-resistant primary tumors in a palliative care setting.

Case 4 in our study involved a patient with good expected long-term survival. Similar to case 1, the most commonly recommended schedule for case 4 was 30 Gy in 10 fractions (67 vs 66%), despite the difference in expected survival. Fractioned intermediate-dose or high-dose regimens were also more commonly recommended for case 4 (24% of the respondents), relative to the recommendation for case 1 with a poor expected survival (9%). However, van der Linden *et al*. [19] evaluated pain relief for BMs in 320 patients who survived > 52 weeks and reported similar pain responses for the single- and multiple-fraction schedules (87% after 8 Gy and 85% after 24 Gy). A single-fraction schedule is the standard palliative treatment for all patients with painful BMs, including patients with an expected favorable prognosis [19]. However, research is still needed to clarify the optimal PORT dose-fractionation schedule for patients with an expected favorable prognosis.

Three-quarters of our respondents recommended that the PORT irradiation field include the ‘entire orthopedic prosthesis’. Optimal PORT irradiation field has not been investigated scientifically in the past, but it was based on clinical experience. In surgical internal fixation, the orthopedic prosthesis is often inserted to stabilize the long bones and is passed through the metastatic sites. It may be suggested that many radiation oncologists preferred to include the ‘entire orthopedic prosthesis’ to eradicate any microscopic residual disease disseminated by the prosthesis. The German Society of Radiation Oncology guideline [20] similarly recommended that metal components used for stabilization should be completely included in the radiation volume. Meanwhie, the ‘entire affected bone’ might be considered too large, whereas the ‘preoperative tumor location’ might be considered too small for the irradiation fields to cover microscopic residual disease disseminated by orthopedic prosthesis. Two retrospective studies of PORT for long BM have also revealed similar results regarding the irradiation field. Townsend *et al*. [8] reported that 21 (84%) of 25 fields included the entire orthopedic prosthesis [8], whereas Drost *et al*. [10] reported that 72 (97.3%) of 74 fields included the entire orthopedic prosthesis. Therefore, it appears to be common practice to include the entire orthopedic prosthesis. Although the objective for long-bone treatment via PORT is unclear, Townsend *et al*. [8] and Adamietz and Wolanczyk [11] have retrospectively determined that PORT efficacy is based on the functional status of the extremities. Another potential objective is to reduce local progression and prevent prosthesis displacement, which may reduce the need for a second surgery [10]. Our results also indicate that many Japanese radiation oncologists (54%) do not recommend 8 Gy in 1 fraction or 20 Gy in 5 fractions for treating long bones in the PORT setting, as they believe longer-fraction RT provides better local control. Nevertheless, the primary objective of palliative RT is generally not local control but benefits related to improved quality of life, reduction in symptoms and overall survival. For instance, Rades *et al*. [21] evaluated 265 patients with metastatic spinal cord compression and reported that long-course RT provided a significantly better 1-year rate of local control, but short- and long-course RT did not provide significantly different results in terms of motor function.

The greatest limitation of the present study was the lack of clinical follow-up data. Furthermore, it is possible that recommendations for hypothetical cases might not truly reflect clinical practice. Nevertheless, the design of clinical trials will need to be guided by an understanding of clinical practice patterns. These trials may be useful for determining the efficacy or optimal dose-fractionation schedule for using PORT to treat BMs involving the long bones.

In conclusion, our results indicate that, in Japan, current practice for using PORT to treat long bone BM typically involves 30 Gy in 10 fractions for the entire orthopedic prosthesis. Furthermore, most Japanese radiation oncologists did not recommend 8 Gy in 1 fraction or 20 Gy in 5 fractions in terms of the local control of the tumor. Fractioned high-dose regimens may be preferred for cases that involve oligometastasis, whereas fractioned low-dose regimens may be preferred for cases with a poor expected survival. Single-fraction RT was not preferred in the palliative setting. These results may be useful for designing clinical trials aimed at determining the efficacy and optimal dose-fractionation schedule for using PORT to treat metastases to the long bones.

## Supplementary Material

JRR_Revised_Supplemental_table1_rraa133

JRR_Revised_Supplemental_table2_rraa133
