# Supplementary material for: Practice patterns for postoperative radiation therapy in patients with metastases to the long bones: a survey of the Japanese Radiation Oncology Study Group
Source: J Radiat Res. 2021 Jan 18;62(2):356–63. doi: 10.1093/jrr/rraa133 (PMC7948830; doi:10.1093/jrr/rraa133)
Supplement: JRR_Revised_Supplemental_table1_rraa133 [file jrr_revised_supplemental_table1_rraa133.docx]

Supplemental table1: Dose fractionation regimens prescribed at JROSG institutions

| Dose-fractionation schedule | Dose per fraction (Gy) | BED10 | n | % |
| --- | --- | --- | --- | --- |
| 8Gy/ 1fr | 8 | 14.4 | 4 | 3 |
| 15Gy/ 3fr | 5 | 22.5 | 1 | 1 |
| 20Gy/ 5fr | 4 | 28 | 29 | 19 |
| 20Gy/ 4fr | 5 | 30 | 1 | 1 |
| 25Gy/ 5fr | 5 | 37.5 | 2 | 1 |
| 24Gy/ 6fr | 4 | 38.4 | 3 | 2 |
| 30Gy/ 10fr | 3 | 39 | 75 | 50 |
| 24Gy/ 3fr | 8 | 43.2 | 1 | 1 |
| 36Gy/ 12fr | 3 | 46.8 | 2 | 1 |
| 37.5Gy/ 15fr | 2.5 | 46.88 | 3 | 2 |
| 30Gy/ 5fr | 6 | 48 | 6 | 4 |
| 40Gy/ 16fr | 2.5 | 50 | 2 | 1 |
| 39Gy/ 13fr | 3 | 50.7 | 5 | 3 |
| 42Gy/ 14fr | 3 | 54.6 | 1 | 1 |
| 40Gy/ 10fr | 4 | 56 | 3 | 2 |
| 45Gy/ 18fr | 2.5 | 56.25 | 1 | 1 |
| 45Gy/ 15fr | 3 | 58.5 | 8 | 5 |
| 50Gy/ 25fr | 2 | 60 | 1 | 1 |
| 48Gy/ 12fr | 4 | 67.2 | 1 | 1 |
| 60Gy/ 24fr | 2.5 | 75 | 1 | 1 |
| 65Gy/ 25fr | 2.6 | 81.9 | 1 | 1 |

*Abbreviation:* BED=biologically effective dose
