# Supplementary material for: Practice patterns for postoperative radiation therapy in patients with metastases to the long bones: a survey of the Japanese Radiation Oncology Study Group
Source: J Radiat Res. 2021 Jan 18;62(2):356–63. doi: 10.1093/jrr/rraa133 (PMC7948830; doi:10.1093/jrr/rraa133)
Supplement: JRR_Revised_Supplemental_table2_rraa133 [file jrr_revised_supplemental_table2_rraa133.docx]

Supplemental table2: Dose fractionation regimens recommended for hypothetical cases

| Dose-fractionation schedule | Dose per fraction (Gy) | Case 1, n (%) | Case 2, n (%) | Case 3, n (%) | | | Case 4, n (%) |
| --- | --- | --- | --- | --- | --- | --- | --- |
| Not recommended | | 2(2) | 2(2) | | 3(3) | 2(2) | |
| 8Gy/ 1fr | 8 | 2(2) | 0 | 0 | | | 2(2) |
| 20Gy/ 5fr | 4 | 16(18) | 0 | 7(8) | | | 3(3) |
| 25Gy/ 5fr | 5 | 1(1) | 0 | 1(1) | | | 0 |
| 24Gy/ 6fr | 4 | 0 | 0 | 1(1) | | | 0 |
| 30Gy/ 10fr | 3 | 60(67) | 40(45) | 47(53) | | | 59(66) |
| 28Gy/ 7fr | 4 | 0 | 0 | 1(1) | | | 0 |
| 36Gy/ 12fr | 3 | 1(1) | 0 | 1(1) | | | 0 |
| 37.5Gy/ 15fr | 2.5 | 0 | 1(1) | 0 | | | 1(1) |
| 39Gy/ 13fr | 3 | 4(4) | 21(24) | 22(25) | | | 12(13) |
| 45Gy/ 18fr | 2.5 | 0 | 0 | 1(1) | | | 1(1) |
| 45Gy/ 15fr | 3 | 2(2) | 12(13) | 5(6) | | | 5(6) |
| 35Gy/ 5fr | 7 | 0 | 2(2) | 0 | | | 0 |
| 50Gy/ 25fr | 2 | 0 | 5(6) | 0 | | | 2(2) |
| 60Gy/ 30fr | 2 | 0 | 5(6) | 0 | | | 1(1) |
| 50Gy/ 10fr | 5 | 0 | 1(1) | 0 | | | 0 |
| Cannot respond | | 1(1) | 0 | | 0 | 1(1) | |
